# 50th anniversary of the Stanford SSRL synchrotron radiation and protein crystallography initiative

**DOI:** 10.1107/S1600577525002917

**Published:** 2026-01-01

**Authors:** John R. Helliwell, Colin Nave, D. Marian Szebenyi

**Affiliations:** a Department of Chemistry University of Manchester ManchesterM13 9PL United Kingdom; b Diamond Light Source Harwell Science and Innovation Campus DidcotOX11 0DE United Kingdom; c MacCHESS, CHESS Cornell University Ithaca USA; RIKEN SPring-8 Center, Japan

**Keywords:** protein crystallography, macromolecular crystallography, synchrotron radiation, XFELs, SSRL Stanford, structural biology

## Abstract

We provide a historical introduction spanning the past 50 years of synchrotron radiation protein crystallography. We then provide a resume of current trends. These help us to celebrate the huge influence that synchrotron radiation, and now X-ray lasers, has had on the scope of protein crystallography. It has also accelerated the development of closely allied methods such as neutron protein crystallography, which has adopted the synchrotron Laue method as its own as well as developing monochromatic and time-of-flight methods. Also, the democratic access to central synchrotron facility beamlines has prompted similarly operated centres of electron cryo-microscopy and micro-electron diffraction. We offer our thoughts on the current trends across this scientific landscape.

## Abstract 2

We provide a historical introduction and our thoughts on the current trends including some based on papers in this special issue of *Journal of Synchrotron Radiation* celebrating the 50th Anniversary of the Stanford SSRL synchrotron radiation and protein crystallography initiative led by Keith Hodgson.

## 1. Introduction

In 2026 it will be the 50th Anniversary of the Stanford Synchrotron Radiation Laboratory (SSRL) synchrotron radiation (SR) and protein crystallography initiative led by Keith Hodgson and published in 1976 in *Proceedings of the National Academy of Sciences of the USA* (hereafter *PNAS*) (Phillips *et al.*, 1976 ▸). A preprint of this publication began circulation in 1975. With the agreement of the IUCr’s *Journal of Synchrotron Radiation* Main Editors and the IUCr Journals’ Editor-in-Chief, we have launched a special issue entitled ‘*The 50th Anniversary of the Stanford SSRL SR and protein crystallography initiative*’ (https://journals.iucr.org/special_issues/2025/ssrlprotein).

### 1.1. On a personal note (JRH)

**Figure 1:**
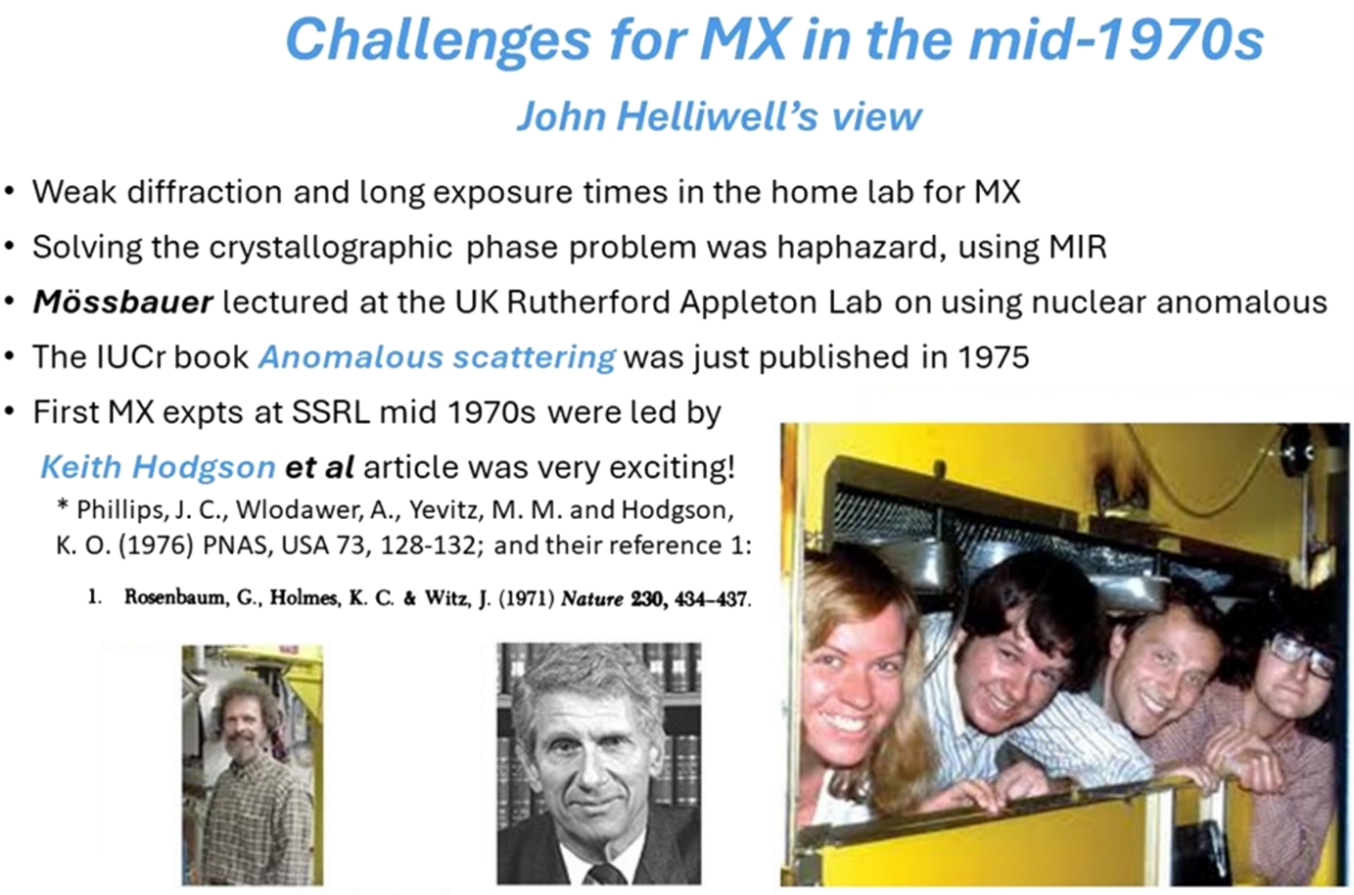
Challenges for macromolecular crystallography in the mid-1970s: my personal view (JRH). Keith Hodgson and his team in the SSRL radiation safety enclosure are in the photograph at right (left to right: Marguerite Yevitz, Keith Hodgson, Alex Wlodawer and James Phillips). Far left is a photograph of Gerd Rosenbaum and at his right is a photograph of Ken Holmes. Abbreviations: MX = macromolecular crystallography; MIR = multiple isomorphous replacement.

When Dorothy Hodgkin shared a preprint of that paper (Phillips *et al.*, 1976 ▸) with me in 1975, when I was a DPhil student at the University of Oxford (1974 to 1977), it changed my whole research career perspectives. I sum the situation up and summarize the challenges for macromolecular crystallography (MX) at that time in Fig. 1 ▸.

Suitably inspired, my first SR experiment commenced in 1976 on the NINA synchrotron at Daresbury; it involved optimizing the anomalous scattering of platinum in my K_2_Pt(CN)_4_ derivative of my DPhil project ‘6-phospho­gluconate de­hydrogenase enzyme protein crystallography structure determination’ (supervisor in Oxford, Margaret Adams). I was helped greatly by my NINA local contact Dr Joan Bordas.

This experiment was soon followed by my use of the LURE synchrotron MX beamline in Paris (established by Roger Fourme) and the EMBL Hamburg SR facilities at DESY (launched by Ken Holmes and Gerd Rosenbaum). Indeed, the seminal article by Rosenbaum, Holmes & Witz (1971 ▸), ‘*Synchrotron Radiation as a Source for X-ray Diffraction*’, at DESY was cited by Phillips *et al.* (1976 ▸) as the first reference of their references list. That paper, also pivotal, described muscle fibre diffraction with SR but did mention SR protein crystallography in a table. The wide influence of this 1971 paper, and how it led to the EMBL Outstation at DESY Hamburg, is described by Holmes & Rosenbaum (1998 ▸). In parallel with the paper by Phillips *et al.* (1976 ▸) was the paper by Harmsen *et al.* (1976 ▸) from DESY, also important, which had a more cautious view of SR protein crystallography, and no coverage of the multiple wavelength phasing method with SR that Hodgson *et al.* initiated and developed (Phillips *et al.*, 1977 ▸, 1979 ▸; Templeton *et al.*, 1980 ▸; Phillips & Hodgson, 1980 ▸). That method was greatly expanded in its applicability by the seleno-me­thio­nine protein production methodology initiated by Wayne Hendrickson (Hendrickson *et al.*, 1990 ▸).

In 1981 I joined the UK’s dedicated SRS leading the protein crystallography there on a 50%/50% joint appointment with Keele University Department of Physics, and subsequently full time at Daresbury from 1983. We made a strong effort to move to electronic detectors jointly with the Rutherford Appleton Laboratory (a multi-wire proportional chamber) and the Laboratory of Molecular Biology Cambridge and Enraf–Nonius (a TV diffractometer of Uli Arndt). Image plate readers came later, from Rigaku and EMBL Hamburg (Jules Hendrix), then CCDs initiated from Princeton (Sol Gruner) and installed at MacCHESS (see below). The pixel-array detector came considerably later, notably from the Swiss PSI Centre and its spin out company Dectris, which dominates the detector provision at beamlines in the modern era.

Within this period, I led the planning of ESRF’s macromolecular crystallography in the mid-1980s and into the 1990s (Aigrain, 1987 ▸). Greatly facilitating the detailing of these plans was the ‘New Rings Workshop’ held at SSRL, that led to a direct research and development collaboration between myself at Daresbury SRS and Keith Hodgson and Britt Hedman at SSRL [see Hedman *et al.* (1985 ▸) and Helliwell (1988 ▸)]. The eventual extensive development of instrumentation and methods for multi-wavelength anomalous dispersion at the SRS, ESRF, CHESS and Elettra facilities in which we participated was described by Cassetta *et al.* (1999 ▸). As a further measure of the global connectedness of the SR research and facility community, Keith Moffat spent a sabbatical year with me at the Daresbury SRS and at the York University Department of Physics, where I had taken up another joint appointment. We worked on various aspects of the SR Laue protein crystallography method. Then in 1994 I spent four and a half months on sabbatical at MacCHESS, Cornell University, with Steve Ealick, then MacCHESS Director, followed by one month at BioCARS with Keith Moffat, by then at the Advanced Photon Source and University of Chicago.

Overall, I described the evolution of SR and the growth of its importance in crystallography in Helliwell (1992 ▸, updated in 2011; see Helliwell, 2011 ▸). There are many new developments in the past ten years since 2012. These are encapsulated in all the articles in this special issue. I pay tribute to the 1976 paper by Keith Hodgson, and his many decades of leadership at SSRL, and the other pioneers at DESY Hamburg (Rosenbaum *et al.*, 1971 ▸) and LURE Paris (Lemonnier *et al.*, 1978 ▸).

### 1.2. On a personal note (DMS)

The Cornell High Energy Synchrotron Source (CHESS) also became a centre for macromolecular crystallography. Cornell had a long history of projecting the importance of SR in X-ray physics including diffraction (Parratt, 1959 ▸). CHESS, constructed in 1978–1980, was one of the first facilities to make use of the parasitic X-rays from an electron–positron collider (CESR, which had been in operation since 1975). Keith Moffat pioneered use of CHESS for macromolecular crystallography, assisting a number of scientists to use the facility beginning in 1981. I recall the excitement of finding that exposures of 24–36 h on a lab source now took only a few minutes at CHESS! In 1984, Moffat secured a grant from NIH to establish the Research Resource MacCHESS (‘Macromolecular crystallography at CHESS’, or ‘son of CHESS’, according to Moffat’s Scottish background), a formalization of the program to support and improve a user facility for structural biologists, and became its first Director. The MacCHESS grant has continued to the present day, and I was part of it from its founding until my retirement in 2022, serving as Director from 2008 to 2022.

The forte of MacCHESS is development of new techniques to facilitate the use of X-rays by structural biologists – largely macromolecular crystallography, but also biological small angle X-ray scattering, BioSAXS. Many of these techniques, developed in the late 20th century, are now standard at synchrotron sources worldwide.

For example, with the intense X-rays from a synchrotron source, it became clear that radiation damage to macromolecular crystals was a serious problem. Cooling the crystals to liquid nitro­gen temperature was the solution, and experiments at CHESS were critical in developing cryocooling techniques. Once it was realized that very rapid cooling was required for success (hence precluding use of a capillary surrounding the crystal), Håkon Hope developed a method of mounting crystals using oil and very thin glass slips (created by blowing a bubble of glass and shattering it) (Hope, 1988 ▸). This method was utilized in tests at CHESS but was quickly supplanted by use of a thin free-standing film of mother liquor enclosed in a loop. The loop mount (Teng, 1990 ▸) was originally developed by T.-Y. Teng, a scientist in Moffat’s lab, as a means of reducing strain on a thin plate crystal, but it proved a boon for cryocooling: easy to make, easy to load with a crystal, and producing very little background scatter (especially after the original gold wire loop was replaced with X-ray transparent nylon). The cryoloop was soon commercialized and became a mainstay of crystallography at synchrotron sources and elsewhere.

Along with other synchrotron facilities, MacCHESS has been active in developing techniques to automate crystal handling, data collection and processing, to the point that now a user need only grow crystals, ship them to the facility, and use a well designed GUI to carry out their experiment. Various beamlines tend to put their own spins on their automation suite, but there has been plenty of ‘cross-pollination’ between them, to which MacCHESS has contributed, particularly in the 1990s and 2000s.

MacCHESS has been key in the development of increasingly sophisticated X-ray detectors for macromolecular crystallography. When Rossmann solved the structure of human rhinovirus at CHESS (Rossmann *et al.*, 1985 ▸), it required processing hundreds of pieces of X-ray film. When imaging plates were developed to replace film, first at Kodak and later by Fuji, they were quickly adopted at MacCHESS to provide digitized diffraction patterns. Then followed extremely productive collaborations with Sol Gruner’s lab and with Area Detector Systems Corp. to test, and integrate into the crystallographic process, CCD-based detectors (Gruner & Ealick, 1995 ▸) and hybrid pixel-array detectors (Rossi *et al.*, 1999 ▸).

Although most data taken at CHESS utilize monochromatic X-rays, MacCHESS pioneered the synchrotron Laue method, beginning in 1984 (Moffat *et al.*, 1984 ▸), as a means of performing very rapid, *i.e.* time-resolved, protein crystallography. Using Laue diffraction in conjunction with a borrowed undulator, CESR running in single-bunch mode and a custom high-speed shutter, it was possible to obtain useful data from a 120 ps pulse of X-rays (Szebenyi *et al.*, 1988 ▸). The Laue method was promptly taken up by Helliwell (1984 ▸) at SRS Daresbury and its users (*e.g.* Hajdu *et al.* 1987 ▸) and extended to neutrons, firstly at the Institut Laue Langevin in Grenoble in France (Habash *et al.*, 1997 ▸).

### 1.3. Protein crystallography at Daresbury 1987 onwards(CN)

By 1987, the protein crystallography facilities on beamlines 9.6 and 7.2 were well established with a high scientific output and beamline 9.5 was being developed for very rapid Laue and rapidly tunable monochromatic experiments, under a collaboration with the Swedish Research Council. Early Laue experiments clarified the capabilities of the technique and acted as a test bed for Laue software developments. For monochromatic data collection, the SRS was an early adopter of data collection at cryogenic temperatures, a technique which allowed the study of weakly diffracting crystals before radiation damage occurred. Daresbury staff investigated radiation damage in protein crystals at cryogenic temperatures in the early 1990s using beamlines 9.7 and 9.5 in Laue mode. The subject of radiation damage in protein crystallography resulted in a regular series of international workshops which has lasted 26 years thus far.

Scientific highlights using these facilities included structures from Oxford of foot and mouth disease virus and time-resolved studies on glycogen phospho­rylase, the structure determination of light harvesting protein (Glasgow and Daresbury) and, in 1994, the structure of F1-ATPase by a team from the MRC Laboratory of Molecular Biology. This work resulted in a Nobel Prize for Chemistry, awarded to John Walker in 1997. There was also increasing interest in the use of the SRS by the pharmaceutical industry and agreements were set up with a variety of companies to provide both beam time and data collection services. By the beginning of the 21st century the protein crystallography facilities at the ESRF were increasingly being used for the most demanding projects. However, even for these, the SRS was being used for initial investigations including screening crystals to identify the best crystallization conditions. One notable example of this is the work on 30S ribosome which led to the Nobel Prize for Chemistry (to Venki Ramakrishnan in 2009). This Nobel Prize was shared with Ada Yonath (also an SRS user) and Tom Steitz.

The research councils (and later the Wellcome Trust) involved in biological and medical sciences were very keen to see that the biology beamlines on the SRS were maintained at a high level and invested additional funds. This resulted in the construction of three new beamlines – 14.1 and 14.2 [available 2000 (Duke *et al.*, 1998 ▸)] and 10.1 [available 2003 (Cianci *et al.*, 2005 ▸)] – on multipole wiggler beamlines, two of which were equipped with multi-wavelength anomalous dispersion (MAD) capabilities. These facilities also acted as a focus for automation and incorporated robotic sample changers.

The Collaborative Computational Project for Protein Crystallography (CCP4) was based at Daresbury during the lifetime of the SRS and good links were developed between these two activities. An informal collaboration was set up with other European synchrotrons and the MRC Laboratory of Molecular Biology with the aim of automating data collection for protein crystallography. Several successful joint grant applications extended this further, funded by UK Research Councils or the European Commission.

After the announcement that the new synchrotron Diamond was to be built on the Rutherford Appleton Laboratory site, investment in the SRS nevertheless continued to sustain a key driver for the new facility. The planned transfer of activities from the SRS to Diamond went well for protein crystallography and the developments in automation and data handling which took place on the SRS formed a good basis for further developments at Diamond.

## 2. Current trends (JRH, CN, DMS)

The pace of developments in protein crystallography on synchrotrons is continuing with new and upgraded sources with higher brightness, matching beamline optics, faster large area detectors, reliable automation and data handling. All of this results in more challenging structural biology projects becoming feasible.

In the past ten years major developments have been the ‘resolution revolution’ for electron cryo-microscopy (‘cryoEM’), the availability of short-pulse-length high-brightness free-electron lasers, and the applications of machine learning software such as *AlphaFold* for protein structure prediction. These have become embedded in the ‘tool box’ needs of the structural biology research community.

In the context of experimental phasing, protein structure prediction by *AlphaFold* has recently had a major impact. Approximately 25 years ago the use of multiple wavelength anomalous dispersion methods for phasing increased significantly, particularly when combined with microbiological production of proteins with direct replacement of me­thio­nine with seleno-me­thio­nine as mentioned earlier. It replaced multiple isomorphous replacement phasing pioneered by Max Perutz and John Kendrew in the 1950s. Later, single-wavelength anomalous dispersion was found to be sufficient in many cases. These methods, when combined with a structural genomics ‘pipeline’ approach, generated many new structures which later meant that the majority of protein structures could then be determined by molecular replacement techniques. At present, models from machine learning methods are often used for molecular replacement, as it is possible to incorporate the exact sequence for the protein of interest. However, anomalous dispersion still has a role, particularly for identifying metal and other heavier atoms within a protein structure.

There is increased activity in neutron macromolecular crystallography with expansions in the suite of global instruments. Neutrons are particularly useful in experimental determination of protonation states of ionisable amino acids (Asp, Glu, His, Arg, Lys), and can resolve enzyme mechanism disputes and definitively determine receptor ligand binding, especially hydrogen bonding. A recent book on the whole topic is in the *Methods of Enzymology* book series (Moody, 2020 ▸).

The availability of many protein structures means that small angle X-ray scattering (SAXS) is increasingly being used both by academics and industry. The technique provides information about complexes formed from proteins of known structure and changes in conditions such as pH, temperature or ligand binding. A recent review is given by Byer *et al.* (2023 ▸).

Increasing emphasis is being given to obtaining images across different length scales, with crystallography and single particle cryoEM giving near atomic resolution. *In situ* structure determination by electron tomography of frozen hydrated cells is making impressive progress (Nogales & Mahamid, 2024 ▸), enabling the atomic resolution structures to be located and perhaps modified within the cell. Because X-rays can penetrate much further than electrons, this information can be carried forward to thicker samples such as tissue (*e.g.* brains, organoids, virus tracking through tissue) and eventually whole organisms. A review of the capabilities of synchrotron X-rays for imaging thick samples has been given by Du *et al.* (2021 ▸), and a comparison of electrons and X-rays for imaging by Du & Jacobsen (2018 ▸). Many synchrotrons are placing increasing emphasis on fragment or other binding studies. These are coming to include binding studies not only at 100 K but also at room temperature, the need for which was pointed out by Halle (2004 ▸) (especially for low occupancies). Such a capability was recently implemented at the SLS (Huang *et al.*, 2022 ▸). At the upgraded synchrotrons, such as ESRF–EBS, flagship beamlines include time-resolved serial crystallography studies (https://www.esrf.fr/id29).

Given all these developments, some implications for synchrotron-based protein crystallography are:

(i) Crystal structure determination of proteins from synchrotron data we think will be a significant activity in the future, both as a basis for ligand binding and time-resolved studies in crystals and to validate and improve on structures from machine learning algorithms.

(ii) In addition to a continued growth in cryoEM we think that the expansion of in cell cryo electron tomography will accelerate.

(iii) Fragment and other binding studies by experimental methods will increasingly be complemented by machine learning algorithms.

(iv) Time-resolved studies will be split between synchrotrons and XFELs.

The huge amount of exciting scientific results from the first experiments in 1975 was not generally anticipated at the time. The questions above are likely to be answered well within the 100th anniversary of the first protein crystallography experiments on a synchrotron but we will have to wait to find out what these answers will be.

A major development is the advent and application of X-ray lasers in protein crystallography. These have been based at the synchrotron centres and have led to a synergistic impact on the synchrotron protein crystallography beamlines. An X-ray laser flash has taken the field from sub-nanosecond pulses of a synchrotron to tens of femtosecond ones. The technique has become known as serial femtosecond crystallography (SFX). Each pulse is so intense that it destroys each tiny crystal that is hit due to its intense X-ray power, but its diffraction occurs before it is destroyed (Neutze *et al.*, 2000 ▸). Therefore, many crystals are needed to cover the full rotation range of a dataset. A description of the genesis of serial crystallography with XFELs was provided by Chapman (2015 ▸) including the various methods used to deliver the sample into the X-ray beam position. The option of a liquid micro-jet was advocated as particularly flexible and that it gave a low X-ray background, suitable for sub-micrometre crystals. Such a jet’s high speed, moving the liquid jet a relatively long distance between X-ray pulses, meant that many crystallites were simply missed by the XFEL pulses. The development of gel extrusion jets being slower has given higher sample usage efficiencies, but at the cost of higher X-ray background in the diffraction image. These flowing systems operate in vacuum. Instead, a fast raster-scan approach of the tiny, focused, X-ray pulse across a single crystal can give an optimum sample consumption efficiency and data collection rate. This method can also be used on a random field of crystals. Sample delivery for XFEL experiments is an area in active development. Schlichting (2015 ▸) provided a helpful overview of the achievements at XFELs in the first five years. A particularly challenging aspect has been the diffraction data processing software and procedures, and their optimization for the new types of experiments. These experiments are intrinsically difficult, but best practices are emerging (Gorel *et al.*, 2021 ▸). An important demonstration was described most recently by Moffat & Lattman (2023 ▸) in their book (see their chapter 3, Box 3B discussion) who give a comparison of SR time-resolved protein crystallography experiments with those carried out at the Linac Coherent Light Source (LCLS) at Stanford involving identical time points for the same biological system (photoactive yellow protein, PYP) in a control experiment. Difference electron density signals were approximately doubled in the LCLS case. The advantage in the LCLS experiments over the synchrotron experiments was due to the much smaller crystals used and their better optical transparency to the laser pump, thus establishing a higher occupancy of the structural intermediates. Moffat & Lattman (2023 ▸) also discuss anxieties about high peak powers of the laser pump initiating protein structure damaging multiphoton effects. They state, ‘*An essential control experiment, a laser power titration should be carried out and should reveal linear dependence of the X-ray signal on pump laser power from a typical sample…. Easy to state but in practice unrealistic to execute fully.’.* This systematic error in the method of photocrystallography is covered in detail by Barends *et al.* (2024 ▸), including showing non-functional structural changes in CO bound to myoglobin. See also the associated *News and Views* article by Neutze & Miller (2024 ▸).

Stimulated by the XFELs introducing serial delivery of samples, synchrotron crystallography beamlines introduced similar approaches collectively known as serial synchrotron crystallography (SSX). This was introduced at EMBL Hamburg by Gati *et al.* (2014 ▸) and elaborated on by Hakanpää *et al.* (2018 ▸) and Stellato *et al.* (2014 ▸), the former for microcrystals grown *in vivo* and the latter examples with a liquid jet of microcrystals. The SSX technique allows a low dose approach to be adopted for both cryogenic and room temperature data collection and allows faster time-resolved measurements. A comparison between SFX and SSX has been given by Mehrabi *et al.* (2021 ▸). The practical details for the fixed target method are described in the article and accompanying six-minute video of Horrell *et al.* (2021 ▸); this involves the use of micro-pipetted micro-crystal slurries into a specially designed chip (*i.e.* samples holder). Most recently, Orlans *et al.* (2025 ▸) have achieved microsecond time-resolution using the ESRF–EBS. This is the first synchrotron beamline capable of delivering high brilliance microsecond X-ray pulses at high repetition rate for the structure determination of biological macromolecules at room temperature.

## 3. A resume of other papers in this special issue

At the time of writing this overview, the articles already show a global representativeness of the synchrotron facilities, including the SSRF, China (Kong *et al.*, 2025); SPring-8 and SACLA, Japan (Yamamoto & Kumasaka, 2025); NSLS II, USA (Aishima *et al.*, 2025); CLS, Canada (Janzen & Fodje, 2025); NSRRC, Taiwan (Chou *et al.*, 2025); DESY, Germany (Oberthür *et al.*, 2025); ESRF–EBS and the Partnership for Structural Biology (Grenoble, Photon and Neutron Campus) (McCarthy *et al.*, 2025); Elettra, Italy (Hegde *et al.*, 2025); KEK Photon Factory, Japan (Matsugaki & Senda, 2025 ▸); MAX IV, Sweden (Gonzalez *et al.*, 2025); BESSY II, Germany (Mueller *et al.*, 2025); LCLS, USA (Mous *et al.*, 2025); SLS and SwissFEL, Switzerland (Wang, 2025); ALS, USA (Ralston *et al.*, 2025); as well as use by industry (*i.e.* AstraZeneca) (Käck & Sjögren, 2025).

We are also delighted to cite the overview article in this 50th anniversary collection of Keith Hodsgon himself (Hodgson, 2026).
